# Functional Characterization of Sex Pheromone Receptors in the Fall Armyworm (*Spodoptera frugiperda*)

**DOI:** 10.3390/insects11030193

**Published:** 2020-03-18

**Authors:** Jin-Meng Guo, Xiao-Long Liu, Si-Ruo Liu, Zhi-Qiang Wei, Wei-Kang Han, Youzhong Guo, Shuang-Lin Dong

**Affiliations:** 1 Key Laboratory of Integrated Management of Crop Disease and Pests, Ministry of Education, College of Plant Protection, Nanjing Agricultural University, Nanjing 210095, China; qdguojinmeng@163.com (J.-M.G.); 2017202032@njau.edu.cn (X.-L.L.); srliu0123@163.com (S.-R.L.); 2018102062@njau.edu.cn (Z.-Q.W.); 2019202059@njau.edu.cn (W.-K.H.); 2 Department of Medicinal Chemistry, Virginia Commonwealth University, Richmond, VA 23298, USA; yguo4@vcu.edu; 3 Institute for Structural Biology, Drug Discovery and Development, Virginia Commonwealth University, Richmond, VA 23219, USA

**Keywords:** sex pheromone, pheromone receptor, *Spodoptera frugiperda*, *Xenopus* oocyte

## Abstract

Pheromone receptors (PRs) found in the antennae of male moths play a vital role in the recognition of sex pheromones released by females. The fall armyworm (FAW), *Spodoptera frugiperda*, is a notorious invasive pest, but its PRs have not been reported. In this report, six candidate PRs (*SfruOR6*, *11*, *13*, *16*, *56* and *62*) suggested by phylogenetic analysis were cloned, and their tissue–sex expression profiles were determined by quantitative real-time PCR (qPCR). All six genes except for *SfruOR6* were highly and specifically expressed in the antennae, with *SfruOR6*, *13* and *62* being male-specific, while the other three (*SfruOR11*, *16* and *56*) were male biased, suggesting their roles in sex pheromone perception. A functional analysis by the *Xenopus* oocyte system further demonstrated that SfruOR13 was highly sensitive to the major sex pheromone component Z9-14:OAc and the pheromone analog Z9,E12-14:OAc, but less sensitive to the minor pheromone component Z9-12:OAc; SfruOR16 responded weakly to pheromone component Z9-14:OAc, but strongly to pheromone analog Z9-14:OH; the other four candidate PRs did not respond to any of the four pheromone components and four pheromone analogs. This study contributes to clarifying the pheromone perception in the FAW, and provides potential gene targets for developing OR-based pest control techniques.

## 1. Introduction

Insect olfaction plays an indispensable role in many important behaviors, such as mating, oviposition and food selection [1]. The olfactory process consists of several major events, including the conversion of volatile compounds into electrical signals at the periphery, integrating the electrical signals in the antennae lobes and ultimately producing behavioral signals in the brain [2]. At the periphery level, the olfaction process involves several classes of proteins, including odorant-binding proteins (OBPs) [3], odorant receptors (ORs) [4], inotropic receptors (IRs) [5], sensory neuron membrane proteins (SNMPs) [6] and odorant-degrading enzymes (ODEs) [7]. Among these proteins, ORs play a central role in determining specificity of olfactory receptor neurons (ORNs) [2,8,9].

ORs are seven transmembrane receptors located on the dendrites of ORNs [10,11]. ORs can be divided into two subtypes, i.e., highly conserved OR coreceptors (ORco) and divergent ligand-specific ORs (ORx). These subtypes interact with each other to form an ORx-ORco complex, which forms odorant-gated ion channels. Pheromone receptors (PRs) refer to members of the OR superfamily that function in the perception of sex pheromones. The first lepidopteran PR was identified in *Bombyx mori* by the *Xenopus* oocyte expression system [12]. Since then, more than 60 PRs have been functionally characterized in over 30 moth species across 10 families using HEK293 cells [13], *Xenopus* oocytes [14], transgenic *Drosophila* [15], and, more recently, the CRISPR/Cas9 system [16]. All of those moths with identified PR, except for *B. mori*, are important agricultural pests, such as *Manduca sexta* [17], *Spodoptera exigua* [18], *Spodoptera litura* [19], *Plutella xylostella* [20,21], *Ostrinia furnacalis* [22] and *Athetis dissimilis* [23].

The fall armyworm (FAW), *Spodoptera frugiperda* (Lepidoptera: Noctuidae), is an important invasive pest that is native to tropical and subtropical regions in North and South America [24,25], and invaded the United States and Canada in the middle of the 19th century [26,27]. In 2016, FAW first appeared in Nigeria and Sao Tome and Principe, and invaded over 40 African countries within two years [28,29,30,31]. In Asia, it invaded India in 2018 and China in early 2019 [32,33]. FAW is polyphagous, feeding on 353 plant species from 76 families [34]. Two host plant strains, the corn strain (C) and the rice strain (R), are remarkably differentiated, having different host plant preferences and being reproductively isolated to a certain extent [35,36,37,38]. The two strains are indistinguishable from each other in external morphology, but differ from one another in terms of the ratio of sex pheromone composition [39]. The extract of the female pheromone glands contains several components, including Z11-16:Ald, Z11-16:OAc, Z11-14:OAc, 11-12:OAc, Z10-14:OAc, Z9-14:OAc, Z9-14:Ald, Z9-12:OAc, 12:OAc, 14:OAc, 16:OAc, Z7-12:OAc and E7-12:OAc [35,40,41,42,43,44], of which four components (Z9-14:OAc, Z11-16:OAc, Z9-12:OAc and Z7-12:OAc) were active in attracting males in the field experiments [41]. These sex pheromones are widely used in the FAW control by large-scale trapping, mating disruption and population monitoring [43,45,46,47]. However, the molecular mechanisms of sex pheromone perception in FAW are mostly unknown.

Previously, six candidate PRs from 69 ORs in the FAW have been suggested by genome sequencing and bioinformatic analysis [48]. In the present study, to functionally characterize the candidate PRs, full length genes were cloned, and expression patterns of these genes were measured between tissues and sexes by quantitative real-time PCR (qPCR). Finally, electrophysiological responses of these candidate PRs to sex pheromones and pheromone analogs were measured using *Xenopus* oocytes and two-electrode voltage clamp. The results provide insights into the mechanisms of sex pheromone perception in the FAW.

## 2. Materials and Methods

### 2.1. Insect Rearing

FAW larvae were fed with an artificial diet [49] and kept at a temperature of 27 ± 1 °C, 65 ± 5% relative humidity (RH), and a photoperiod of 14:10 h (Light: Dark). Male and female pupae were positioned separately in cages for eclosion. Adults were provided with a 10% honey solution.

### 2.2. RNA Extraction and cDNA Synthesis

Total RNA was isolated from collected tissues using Trizol Reagent (Invitrogen, Carlsbad, CA, USA) according to the manufacturer’s protocol. The quality and concentration of RNA were verified by NanoDrop-2000 (Thermo Scientific, Waltham, MA, USA). The 260/280 ratios were all 1.8–2.0. The first single-strand cDNAs were synthesized from 1µg total RNA using HiScript^®^ III RT SuperMix for qPCR (+gDNA wiper) (Vazyme, Nanjing, China) according to the provided protocol. The 4× gDNA wiper Mix contained in the kit completely removes residual genomic DNAs from the RNA template, ensuring more reliable quantitative results. The control PCR was also conducted to confirm that there was no gDNA contamination in the RNA samples. The prepared cDNA templates were stored at −20 °C prior to use.

### 2.3. Gene Cloning

The six candidate PR genes (*SfruOR6*, *11*, *13*, *16*, *56*, and *62*) and *SfruORco* were cloned with specific primers (Table S1) designed by Primer5.0 (PREMIER Biosoft International, CA, USA) according to reported sequences in BIPAA [48]. The open-reading frames (ORFs) of these six genes were predicted using the ORF Finder (National Center for Biotechnology Information). The PCR was performed in 25 µL containing 12.5 µL of 2 × Phanta Max Master Mix (super fidelity), 9.5 µL of ddH_2_O, 1 µL of cDNA template, and 1 µL forward and reverse primers (10 µM). The PCR conditions were 95 °C for 3 min; 35 cycles of 95 °C for 15 s, 55 °C for 15 s, 72 °C for 90 s; 72 °C for 8 min. PCR products were run on a 1.2% agarose gel, and the band was recovered and purified by AxyPrep™ DNA Gel Extraction Kit (Axygen, Suzhou, China). Purified PCR products were cloned in the pEASY^®^-Blunt3 Cloning Vector (TransGen Biotech, Beijing, China) and then transformed into Trans1-T1 Phage Resistant Chemically Competent Cells (TransGen Biotech, Beijing, China). The transformants were screened on LB-Agar plates containing 100 μg mL^−1^ ampicillin. The positive clones were sequenced by the company Tongyong (Chuzhou, China).

### 2.4. Phylogenetic Analysis

Transmembrane domains of the six candidate PRs were predicted by TMHMM Server Version 2.0 (DTU Bioinformatics Technical University of Denmark, Copenhagen, Denmark), and the multiple sequence alignment and identity calculation were performed by the DNAMAN 8.0 software (Lynnon Biosoft, San Ramon, CA, USA). To construct the phylogenetic tree, ORs from four *Spodoptera* species, i.e., *S. litura* [19,50], *S. exigua* [18], *S. frugiperda* and *Spodoptera littoralis* [51], were used. The amino acid sequences were aligned by MAFFT version 7 [52]. Phylogenetic trees were constructed using RAxML version 8 [53] with JTT model predicted by ProtTest 3 [54]. Node support was assessed using a bootstrap method based on 1000 replicates.

### 2.5. Tissue Expression Profiles by qPCR

The adult tissues (80 antennae, 80 proboscises, 10 thoraxes, 10 abdomens, 80 legs, and 30 wings) of each sex were dissected from 2-day-old virgin moths and between the 6th and 8th hour of the dark period, and were immediately frozen in liquid nitrogen and stored at −80 °C for use. Total RNA was extracted and cDNA templates were synthesized as mentioned above. The primers used for qPCR were designed by Beacon Designer 8.0 (PRIMER Biosoft International, Palo Alto, CA, USA) (Table S1). The qPCR was performed with ChamQTM Universal SYBR^®^ qPCR Master Mix (Vazyme, Nanjing, China) and QuantStudio™ 6 Flex Real-Time PCR System (Applied Biosystems, Foster City, CA, USA) in 20 μL reactions containing 10 μL of 2 × ChamQ Universal SYBR^®^ qPCR Master Mix, 0.4 µL forward and 0.4 µL reverse primers (10 µM), 1 µL cDNA template and 8.2 µL nuclease-free water. The thermal cycling procedures were as follows: 95 °C for 30 s, 40 cycles of 95 °C for 5 s and 60 °C for 34 s. The *RPL32* and *EF-1α* gene were used as the reference genes to standardize the target gene expression [55].

For each tissue, three biological replications were measured with three technical replicates for each replicate. Gene expression levels were analyzed using the 2 ^−ΔΔCT^ method [56].

### 2.6. Vector Construction and cRNA Synthesis

The ORFs of six candidate PR genes and *SfruORco* gene were amplified using primers with a cutting site of *EcoRI* or *XbaI* (Table S1), and were then cloned into pGH19 vector that was double-digested with *EcoRI* and *XbaI*, using the ClonExpress^®^ One Step Cloning Kit (Vazyme, Nanjing, China). The plasmid was extracted by the Miniprep method and purified with phenol-chloroform-isoamyl alcohol. The purified plasmid was linearized with a restriction enzyme (*NotI*/*NdeI*) and used as templates to synthesize cRNAs by using T7 polymerase of mMESSAGE mMACHINE^®^ T7 Kit (Thermo Fisher Scientific, Waltham, MA, USA). The purified cRNAs were diluted with nuclease-free water at a concentration of 2 µg/µL and stored at −80 °C until use.

### 2.7. Receptor Expression in Xenopus Oocytes and Two Electrode Voltage Clamp Electrophysiological Recordings

The six candidate PRs were expressed in *Xenopus* oocytes and ligand sensitivity was detected using two electrode voltage clamps as previously reported [23]. Eight chemicals (four pheromone components and four pheromone analogs) were used to determine the ligand response profiles. All the chemicals were purchased from Nimrod Inc (Changzhou, China) (purity >95%), and stored as specified by the manufacturer. The 1 M stock solutions in dimethyl sulfoxide (DMSO) were prepared and stored at −20 °C. The stock solution was diluted in Ca^2+^-free standard oocyte saline (SOS) buffer (100 mM NaCl, 2 mM KCl, 1.8 mM CaCl_2_, 1 mM MgCl_2_, 5 mM HEPES, pH 7.6) before the experiments. All chemicals were freshly prepared for the experiments. For each chemical, 5–7 oocytes (replicates) were tested in the screening tests and in the dose-response tests. Oocytes injected with sterilized ultrapure H_2_O were used as controls.

## 3. Results

### 3.1. Gene Cloning and Phylogenetic Analysis

**Figure 1:**
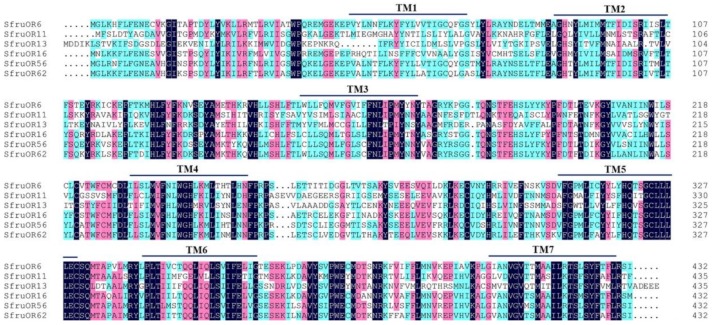
Alignment of the amino acid sequences of the six candidate PRs. The approximate positions of the seven predicted transmembrane domains (TM1–TM7) are indicated by black lines.

**Figure 2:**
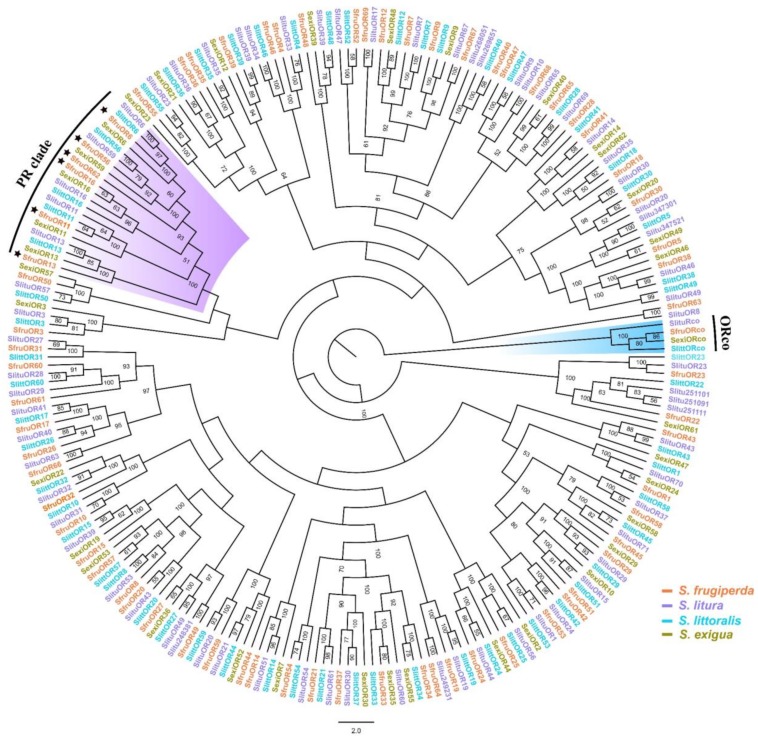
Phylogenetic analysis of ORs from four *Spodoptera* species. Sfru: *S. frugiperda*, Slitu: *S. litura*, Slitt: *S. littoralis* and Sexi: *S. exigua*. The six candidate PR genes in *S. frugiperda* are all clustered in the PR clade, and are denoted with black stars.

Three to five positive clones for each OR gene were sequenced, showing consistent results in different clones. Six candidate PRs (*SfruOR6*, *11*, *16*, *56,* and *62*) contain complete ORFs of 1299, 1308, 1308, 1299, 1299, and 1299 bp, which encode 432, 435, 435, 432, 432, and 432 amino acid residues, respectively. These candidate PRs all contain putative seven transmembrane domains (TMDs) (Figure 1). In the phylogenetic analysis, these candidate PRs were all clustered into the same branch with other Lepidoptera PRs, and were distinct from the general odorant receptor branch (Figure 2).

Except for SfruOR11, the amino acid sequences of other five candidate PRs obtained in the present study were different in 1–7 amino acids from the reported sequences by genomic sequence analyses (Figure S1). In our study, a DNA polymerase of high fidelity (2× Phanta Max Master Mix) was used to amplify the OR genes. The resulting sequences among different clones were the same, suggesting that these OR sequences are correct. Therefore, the OR sequences obtained in the present study were used in the following experiments.

### 3.2. Tissue-sex Expression Profiles

**Figure 3:**
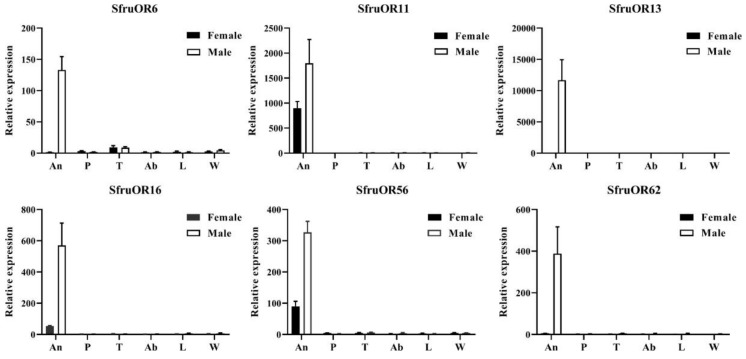
Tissue-sex expression profiles of six candidate SfruPR genes assessed by qPCR. An, antennae; P, proboscises; T, thoraxes; Ab, abdomens; L, legs; W, wings.

Transcription levels of the six candidate PRs were measured by qPCR across different tissues and sexes (Figure 3). All six PR genes were expressed in higher levels in male antennae than female antennae, and *SfruOR6*, *SfruOR13,* and *SfruOR62* were male specific. All genes were undetectable or had very low expression levels in other tissues including proboscises, legs, abdomen, and wings. In addition, *SfruOR13* had the highest expression among the six ORs in the antennae of male FAW.

### 3.3. Functional Characterization of Six Candidate PRs

*Xenopus* oocytes were used to express the PR genes for functional study. Each of the six PRs were co-expressed with the coreceptor SfruORco, and screened for responsiveness to a panel of pheromone components (Z9-14:OAc, Z11-16:OAc, Z7-12:OAc, and Z9-12:OAc) and analogs (Z9,E12-14:OAc, Z9,E11-14:OAc, Z9-14:OH, and Z11-16:Ald). First, the ligand activity of each compound was measured at a screening concentration of 10^−4^ M; then, the dose-response relationship was determined for the active compounds with a range of ligand concentrations (10^−8^–10^−3^ M).

**Figure 4:**
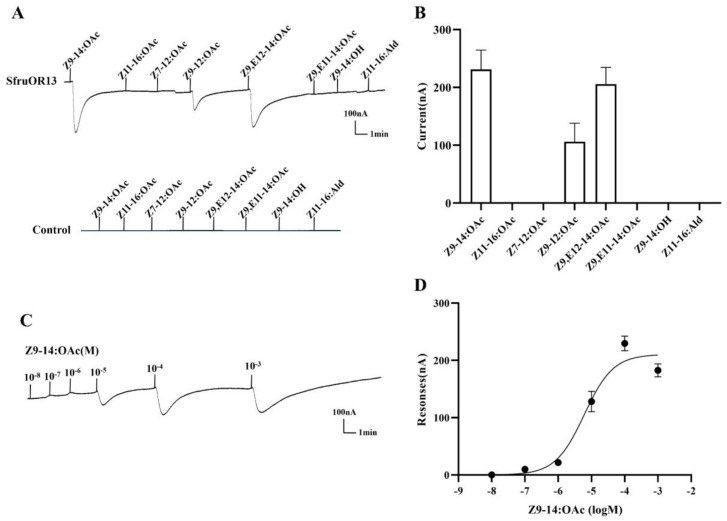
Responses of *Xenopus* oocytes, co-expressing SfruOR13/ORco, to sex pheromone components and analogs. (**A**) Inward current values of oocytes, injected with SfruOR13/ORco (upper) and with buffer (lower), induced by compounds at concentrations of 10^−4^ M. (**B**) Response profile of *Xenopus* oocytes expressing the SfruOR13/ORco complex. Error bars indicate SEM (n = 6). (**C**) Responses of *Xenopus* oocytes, co-expressing SfruOR13/ORco, to Z9-14:OAc at varying concentrations. (**D**) Dose-response curve of *Xenopus* oocytes, co-expressing SfruOR13/ORco, to Z9-14:OAc. EC_50_ value was calculated to be 6.078 × 10^−6^ M. Error bars indicate SEM (n = 5).

**Figure 5:**
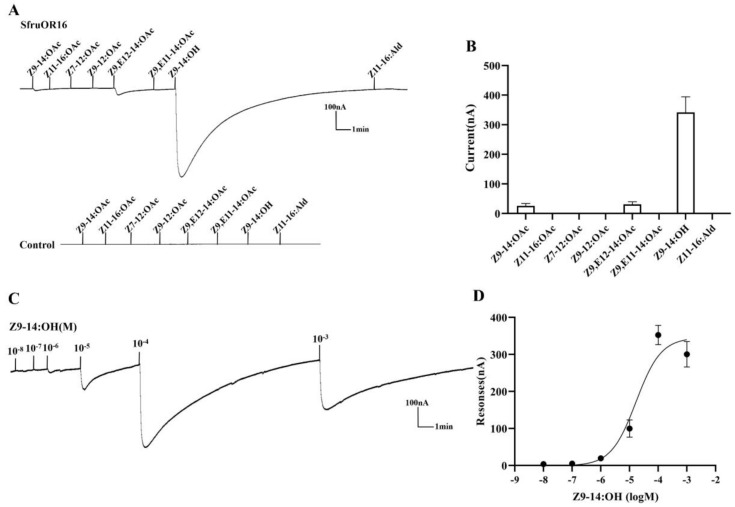
Responses of *Xenopus* oocytes, co-expressing SfruOR16/ORco, to sex pheromone components and analogs. (**A**) Inward current responses of *Xenopus* oocytes, injected with SfruOR16/ORco (upper) and with buffer (lower), induced compounds at concentrations of 10^−4^ M. (**B**) Response profile of *Xenopus* oocytes expressing SfruOR16/ORco. Error bars indicate SEM (n = 6). (**C**) Responses of *Xenopus* oocytes, co-expressing with SfruOR16/ORco, to Z9-14:OH at varying concentrations. (**D**) Dose-response curves of *Xenopus* oocytes, co-expressing SfruOR16/ORco, to Z9-14:OH. EC_50_ value was calculated to be 1.691 × 10^−5^ M. Error bars indicate SEM (n = 5).

The screening test showed that SfruOR13 was activated by two pheromone components, i.e., Z9-14:OAc and Z9-12:OAc, as well as a pheromone analog Z9,E12-14:OAc, with the current values of 231 nA, 106 nA, and 205 nA, respectively (Figure 4A,B). According to the dose-response experiments, the EC_50_ of Z9-14:OAc was calculated to be 6.078 × 10^−6^ M (Figure 4C,D). A very strong response was observed from SfruOR16 when bound to the pheromone analog Z9-14:OH (342 nA), while weak responses were displayed when bound to pheromone component Z9-14:OAc (26 nA) and pheromone analog Z9,E12-14:OAc (31 nA) in the screening test (Figure 5A,B). An additional dose-response experiment showed an EC_50_ value of 1.691 × 10^−5^ M to Z9-14:OH (Figure 5C,D).

The other four candidate PRs (SfruOR6, 11, 56 and 62) failed to produce responses (inward current < 10 nA) to any of the tested pheromone compounds and analogs (Figure S2).

## 4. Discussion

PRs play a vital role in determining the specificity of the sex pheromone communication system between male and female moths, which is considered an important mechanism of interspecies isolation [57,58]. Being an important invasive pest, and especially after the rapid spread to Africa and Asia in recent years, FAW has attracted increasing attention of researchers worldwide. To explore the molecular mechanisms of the sex pheromone perception in this pest, a repertoire of 69 OR genes was identified by informatic analyses of the genomic sequence [48,59], and six candidate PRs were suggested by phylogenetic analysis [48]. In the present study, functional analyses were conducted on these six candidate PRs using the *Xenopus* oocyte system; this confirmed that two of these putative PRs (OR13 and OR16) displayed sensitivity to one or more sex pheromone components, and thus, are likely acting as PRs within FAW.

Of these two PRs, SfruOR13 (displaying the highest expression levels in the antennae of males) responds to the major sex pheromone component Z9-14:OAc and the minor component Z9-12:OAc. This indicates that the major PR (the most highly expressed PR) responds to the major pheromone component, similar to those in some other moths such as *Helicoverpa armigera* [60], *P. xyllostella* [21], and *A. dissimilis* [23]. In addition to SfruOR13, SfruOR16 also responds to the major component, albeit with low sensitivity, indicating the importance of the major component. Having two ORs for the same odorants, but with different sensitivities, would allow for a precise coding of the odorant quantity over a large range of concentrations, which is critical for insects to successfully discriminate specific odorant mixtures [61]. However, we noticed that the two other minor pheromone components (Z11-16:OAc and Z7-12:OAc) failed to activate any of the six ORs tested, suggesting that some other ORs outside the PR clade are responsible for their detection. Recently, it was reported that in *S. littoralis*, SlittOR5, a member of the general odorant receptor clade, responds to the sex pheromone component Z9,E11-14:OAc, rather than one of the receptors in the PR clade [62].

**Figure 6:**
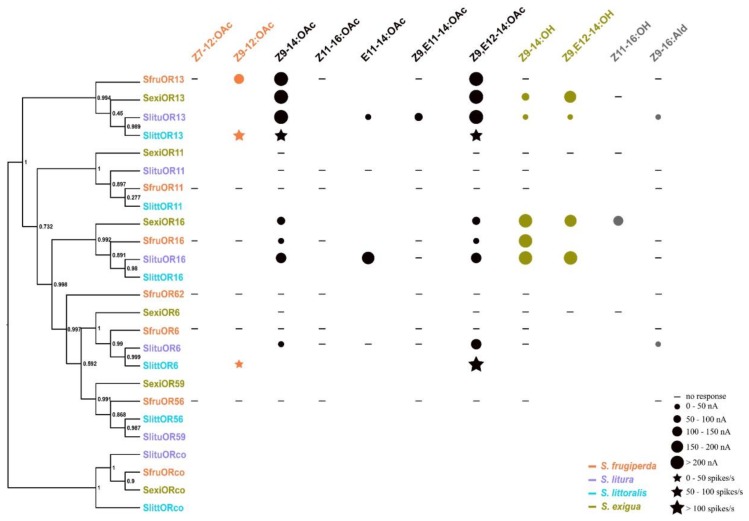
Response profiles of PRs from four *Spodoptera* species. The left part is the phylogenetic tree of candidate PRs from the four species. The right is the response profiles of these candidate PRs (*S. frugiperda* (Figure 4 and Figure 5), *S. exigua* [18], *S. littoralis* [15,69], and *S. litura* [19]). The dots represent the responses obtained by the *Xenopus* oocytes system, and the stars represent responses by in vivo heterologous expression in *Drosophila* OSNs. The blanks indicate compounds that are not tested. The color of the pheromone component corresponds to the color of the insect species to which the sex pheromone belongs, and the black refers to the components shared by two or more species. Z9-14:OAc is shared by the four *Spodoptera* species; Z9,E12-14:OAc is shared by *S. litura*, *S. littoralis* and *S. exigua*; Z9,E11-14:OAc and E11-14:OAc are shared by *S. litura* and *S. littoralis*. Z11-16:OH and Z9-16:Ald in gray are not sex pheromones for the four *Spodoptera* species.

In addition to pheromone components, SfruOR13 also responds to pheromone analog Z9,E12-14:OAc, and SfruOR16 to pheromone analog Z9-14:OH. These two pheromone analogs are actually the sex pheromone components of *S. exigua*, and were used for male trapping in a ratio of 9:1 in the field [63,64,65].Z9,E12-14:OAc is also a minor sex pheromone component in another *Spodoptera* species *S. litura* [66]. In its native range in the Americas, the FAW share a similar geographic distribution with *S. exigua* [67]; thus, SfruOR13 and SfruOR16 are possibly involved in interspecific recognition between these two *Spodoptera* species. Very recently, we showed that sympatric *S. exigua* and *S. litura* can both perceive specific components, reinforcing behavioral isolation between the two species [68]. To analyze the correlation between PR amino acid sequence identity and the ligand specificity, we summarized the results of previous studies from these four *Spodoptera* species [18,19,69], and a clear correlation was shown (Figure 6). For example, four OR13 homologs, sharing 93.15% identity, all have a strong response to Z9-14:OAc and Z9,E12-14:OAc. However, there are exceptions, particularly for OR6 homologs to Z9,E12-14:OAc. SlituOR6 and SlittOR6 are highly sensitive to this ligand, while OR6s in the other two *Spodoptera* species are insensitive, suggesting that minor differences in amino acids are crucial for ligand sensitivity. Clarifying these key amino acids could be of great importance for developing behavioral inhibitors of high activity, which could be used for pest control.

Four of the candidate PRs (SfruOR6, 11, 56, and 62) do not respond to any sex pheromone components tested. This is similar to the results in some previous studies with other moths [14,18,19,21,23,60]. One explanation is that they may tune to other, as yet undescribed, FAW sex pheromone components or sex pheromone components of other species that are not on the list of chemicals under test [70,71]. The other reason might be that the heterologous expression system used in the present study, needs other factors present (such as OBPs or SNMPs) for the function of these candidate PRs. In the *Xenopus* oocyte system, the sensitivity and specificity of pheromones is greatly improved in the presence of certain PBPs [21,72,73], and of SNMP1 [74,75]. Whether PBPs or SNMPs are required for the function of these candidate PRs needs to be further explored.

## 5. Conclusions

To functionally characterize PRs of *S. frugiperda*, we cloned the full-length cDNAs of six candidate PR genes, suggested by the phylogenetic analysis, and determined their specific or biased expression in male antennae. Functional characterization using the *Xenopus* oocyte system demonstrated that SfruOR13 and SfruOR16 were PRs for the sex pheromone component Z9-14:OAc and Z9-12:OAc, while the other four candidate PRs did not respond to any of the four pheromone components. PRs for two other pheromone components (Z11-16:OAc and Z7-12:OAc) need to be revealed by further study. Our study sheds light on the mechanisms underlying sex pheromone perception, and also provides potential targets with which to develop PR-based pest control techniques.

## Supplementary Materials

The following are available online at https://www.mdpi.com/2075-4450/11/3/193/s1, Figure S1: Alignment of the reported candidate PR sequences obtained by genome sequence analysis (GSfruORs) with the sequences cloned in the present study (CSfruORs), Figure S2: Responses of *Xenopus* oocytes co-expressing SfruOR6/ORco (A), SfruOR11/ORco (B), SfruOR56/ORco (C) and SfruOR62/ORco (D) to stimulation with pheromone components and analogs (10^−4^ M), Table S1: Primers used in this study.

## References

[1] Hildebrand J.G., Shepherd G.M. (1997). Mechanisms of olfactory discrimination: Converging evidence for common principles across phyla. Annu. Rev. Neurosci..

[2] Leal W.S. (2013). Odorant reception in insects: Roles of receptors, binding proteins, and degrading enzymes. Annu. Rev. Entomol..

[3] Vogt R.G., Riddiford L.M. (1981). Pheromone binding and inactivation by moth antennae. Nature.

[4] Buck L., Axel R. (1991). A novel multigene family may encode odorant receptors: A molecular basis for odor recognition. Cell.

[5] Benton R., Vannice K.S., Gomez-Diaz C., Vosshall L.B.J.C. (2009). Variant ionotropic glutamate receptors as chemosensory receptors in *Drosophila*. Cell.

[6] Rogers M.E., Sun M., Lerner M.R., Vogt R.G. (1997). Snmp-1, a novel membrane protein of olfactory neurons of the silk moth *Antheraea polyphemus* with homology to the CD36 family of membrane proteins. J. Biol. Chem..

[7] Ishida Y., Leal W.S. (2008). Chiral discrimination of the Japanese beetle sex pheromone and a behavioral antagonist by a pheromone-degrading enzyme. Proc. Natl. Acad. Sci. USA.

[8] Hallem E.A., Ho M.G., Carlson J.R. (2004). Themolecular basis of odor coding in the *Drosophila* antenna. Cell.

[9] Fleischer J., Pregitzer P., Breer H., Krieger J. (2018). Access to the odor world: Olfactory receptors and their role for signal transduction in insects. Cell. Mol. Life Sci..

[10] Vosshall L.B., Amrein H., Morozov P.S., Rzhetsky A., Axel R. (1999). A spatial map of olfactory receptor expression in the *Drosophila* antenna. Cell.

[11] Butterwick J.A., Del Mármol J., Kim K.H., Kahlson M.A., Rogow J.A., Walz T., Ruta V. (2018). Cryo-EM structure of the insect olfactory receptor Orco. Nature.

[12] Sakurai T., Nakagawa T., Mitsuno H., Mori H., Endo Y., Tanoue S., Yasukochi Y., Touhara K., Nishioka T. (2004). Identification and functional characterization of a sex pheromone receptor in the silkmoth *Bombyx mori*. Proc. Natl. Acad. Sci. USA.

[13] Forstner M., Breer H., Krieger J. (2009). A receptor and binding protein interplay in the detection of a distinct pheromone component in the silkmoth *Antheraea polyphemus*. Int. J. Biol. Sci..

[14] Wang G., Vásquez G.M., Schal C., Zwiebel L.J., Gould F. (2010). Functional characterization of pheromone receptors in the tobacco budworm *Heliothis virescens*. Insect Mol. Biol..

[15] Montagné N., Chertemps T., Brigaud I., François A., François M.C., De Fouchier A., Lucas P., Larsson M.C., Jacquin-Joly E. (2012). Functional characterization of a sex pheromone receptor in the pest moth *Spodoptera littoralis* by heterologous expression in *Drosophila*. Eur. J. Neurosci..

[16] Chang H., Liu Y., Ai D., Jiang X., Dong S., Wang G. (2017). A pheromone antagonist regulates optimal mating time in the moth *Helicoverpa armigera*. Curr. Biol..

[17] Große-Wilde E., Stieber R., Forstner M., Krieger J., Wicher D., Hansson B.S. (2010). Sex-specific odorant receptors of the tobacco hornworm *Manduca sexta*. Front. Cell. Neurosci..

[18] Liu C., Liu Y., Walker W.B., Dong S., Wang G. (2013). Identification and functional characterization of sex pheromone receptors in beet armyworm *Spodoptera exigua* (Hübner). Insect Biochem. Mol. Biol..

[19] Zhang J., Yan S., Liu Y., Jacquin-Joly E., Dong S., Wang G. (2014). Identification and functional characterization of sex pheromone receptors in the common cutworm (*Spodoptera litura*). Chem. Senses.

[20] Liu Y., Liu Y., Jiang X., Wang G. (2018). Cloning and functional characterization of three new pheromone receptors from the diamondback moth, *Plutella xylostella*. J. Insect Physiol..

[21] Sun M., Liu Y., Walker W.B., Liu C., Lin K., Gu S., Zhang Y., Zhou J., Wang G. (2013). Identification and characterization of pheromone receptors and interplay between receptors and pheromone binding proteins in the diamondback moth, *Plutella xyllostella*. PLoS ONE.

[22] Liu W., Jiang X.-C., Cao S., Yang B., Wang G.-R. (2018). Functional studies of sex pheromone receptors in Asian corn borer *Ostrinia furnacalis*. Front. Physiol..

[23] Liu X.-L., Sun S.-J., Khuhro S.A., Elzaki M.E.A., Yan Q., Dong S.-L. (2019). Functional characterization of pheromone receptors in the moth *Athetis dissimilis* (Lepidoptera: Noctuidae). Pestic. Biochem. Physiol..

[24] Sparks A.N. (1979). A review of the biology of the fall armyworm. Fla. Entomol..

[25] Capinera J.L. (1999). Fall Armyworm, Spodoptera frugiperda (J.E. Smith) (Insecta: Lepidoptera: Noctuidae).

[26] Johnson J.S. (1987). Migration and the life history strategy of the fall armyworm, *Spodoptera frugiperda* in the western hemisphere. Int. J. Trop. Insect Sci..

[27] Early R., González-Moreno P., Murphy S., Day R. (2018). Forecasting the global extent of invasion of the cereal pest *Spodoptera frugiperda*, the fall armyworm. NeoBiota.

[28] Roger D., Phil A., Melanie B., Tim B., Victor C., Matthew C., Yelitza C., Natalia C., Regan E., Julien G. (2017). Fall armyworm: Impacts and implications for Africa. Outlooks Pest Manag..

[29] Rwomushana I., Bateman M., Beale T., Beseh P., Cameron K., Chiluba M., Clottey V., Davis T., Early R., Godwin J. (2018). Fall Armyworm: Impacts and Implications for Africa.

[30] Goergen G., Kumar P.L., Sankung S.B., Togola A., Tamò M., Luthe D.S. (2016). First report of outbreaks of the fall armyworm *Spodoptera frugiperda* (J.E. Smith) (Lepidoptera, Noctuidae), a new Alien invasive pest in west and central Africa. PLoS ONE.

[31] Cock M.J.W., Beseh P.K., Buddie A.G., Cafá G., Crozier J. (2017). Molecular methods to detect *Spodoptera frugiperda* in Ghana, and implications for monitoring the spread of invasive species in developing countries. Sci. Rep..

[32] Sharanabasappa D., Kalleshwaraswamy C.M., Asokan R., Swamy H.M.M., Maruthi M.S., Pavithra H.B., Hegde K., Navi S., Prabhu S.T., Goergen G. (2018). First report of the fall armyworm, *Spodoptera frugiperda* (J.E. Smith) (Lepidoptera: Noctuidae), an alien invasive pest on maize in India. Pest Manag. Hortic. Ecosyst..

[33] Wu Q., Jiang Y., Wu K. (2019). Analysis of migration routes of the fall armyworm *Spodoptera frugiperda* (J.E. Smith) from Myanmar to China. Plant Prot..

[34] Montezano D., Specht A., Sosa-Gómez D., Roque-Specht V., Sousa-Silva J., Paula-Moraes S., Peterson J., Hunt T. (2018). Host plants of *Spodoptera frugiperda* (Lepidoptera: Noctuidae) in the Americas. Afr. Entomol..

[35] Pashley D.P. (1986). Host-associated genetic differentiation in fall armyworm (Lepidoptera: Noctuidae): A sibling species complex?. Ann. Entomol. Soc. Am..

[36] Dumas P., Legeai F., Lemaitre C., Scaon E., Orsucci M., Labadie K., Gimenez S., Clamens A.-L., Henri H., Vavre F. (2015). *Spodoptera frugiperda* (Lepidoptera: Noctuidae) host-plant variants: Two host strains or two distinct species?. Genetica.

[37] Saldamando-Benjumea C.I., Estrada-Piedrahíta K., Velásquez-Vélez M.I., Bailey R.I. (2014). Assortative mating and lack of temporality between corn and rice strains of *Spodoptera frugiperda* (Lepidoptera, Noctuidae) from Central Colombia. J. Insect Behav..

[38] Velásquez-Vélez M., Saldamando-Benjumea C., Ríos-Diez J. (2011). Reproductive isolation between two populations of *Spodoptera frugiperda* (Lepidoptera: Noctuidae) collected in corn and rice fields from Central Colombia. Ann. Entomol. Soc. Am..

[39] Groot A.T., Marr M., Heckel D.G., Schöfl G. (2010). The roles and interactions of reproductive isolation mechanisms in fall armyworm (Lepidoptera: Noctuidae) host strains. Ecol. Entomol..

[40] Pashley D.P. (1988). Quantitative genetics, development, and physiological adaptation in host strains of fall armyworm. Evolution.

[41] Groot A.T., Marr M., Schöfl G., Lorenz S., Svatos A., Heckel D.G. (2008). Host strain specific sex pheromone variation in *Spodoptera frugiperda*. Front. Zool..

[42] Sekul A.A., Sparks A.N. (1967). Sex pheromone of the fall armyworm moth: Isolation, identification, and synthesis. J. Econ. Entomol..

[43] Tumlinson J., Mitchell E., Teal P., Heath R., Mengelkoch L. (1986). Sex pheromone of fall armyworm, *Spodoptera frugiperda* (J.E. Smith). J. Chem. Ecol..

[44] Descoins C., Silvain J., Lalanne-Cassou B., Cheron H. (1988). Monitoring of crop pests by sexual trapping of males in Guadeloupe and Guyana. Agric. Ecosyst. Environ..

[45] Mitchell E.R. (1979). Monitoring adult populations of the fall armyworm. Fla. Entomol..

[46] Pair S., Raulston J., Sparks A., Sims S., Sprenkel R., Douce G., Carpenter J. (1989). Pheromone traps for monitoring fall armyworm, *Spodoptera frugiperda* (Lepidoptera: Noctuidae), populations. J. Entomol. Sci..

[47] Mitchell E.R., McLaughlin J.R. (1982). Suppression of mating and oviposition by fall armyworm and mating by corn earworm in corn, using the air permeation technique. J. Econ. Entomol..

[48] Gouin A., Bretaudeau A., Nam K., Gimenez S., Aury J.-M., Duvic B., Hilliou F., Durand N., Montagné N., Darboux I. (2017). Two genomes of highly polyphagous lepidopteran pests (*Spodoptera frugiperda*, Noctuidae) with different host-plant ranges. Sci. Rep..

[49] Huang C., Zhu L., Ni J., Chao X. (2002). A method of rearing the beet armyworm *Spodoptera exigua*. Chin. Bull. Entomol..

[50] Cheng T., Wu J., Wu Y., Chilukuri R.V., Huang L., Yamamoto K., Feng L., Li W., Chen Z., Guo H. (2017). Genomic adaptation to polyphagy and insecticides in a major East Asian noctuid pest. Nat. Ecol. Evol..

[51] Walker W.B., Roy A., Anderson P., Schlyter F., Hansson B.S., Larsson M.C. (2019). Transcriptome analysis of gene families involved in chemosensory function in *Spodoptera littoralis* (Lepidoptera: Noctuidae). BMC Genom..

[52] Katoh K., Standley D.M. (2013). MAFFT multiple sequence alignment software version 7: Improvements in performance and usability. Mol. Biol. Evol..

[53] Stamatakis A. (2014). RAxML version 8: A tool for phylogenetic analysis and post-analysis of large phylogenies. Bioinformatics.

[54] Darriba D., Taboada G.L., Doallo R., Posada D. (2011). ProtTest 3: Fast selection of best-fit models of protein evolution. Bioinformatics.

[55] Huot L., George S., Girard P.-A., Severac D., Nègre N., Duvic B. (2019). *Spodoptera frugiperda* transcriptional response to infestation by Steinernema carpocapsae. Sci. Rep..

[56] Livak K., Schmittgen T. (2000). Analysis of rrelative gene expression data using real-time quantitative PCR and the 2^-^^ΔΔCt^ method. Methods.

[57] Groot A.T., Horovitz J.L., Hamilton J., Santangelo R.G., Schal C., Gould F. (2006). Experimental evidence for interspecific directional selection on moth pheromone communication. Proc. Nat. Acad. Sci. USA.

[58] Kurtovic A., Widmer A., Dickson B.J. (2007). A single class of olfactory neurons mediates behavioural responses to a *Drosophila* sex pheromone. Nature.

[59] Liu H., Lan T., Fang D., Gui F., Wang H., Guo W., Cheng X., Chang Y., He S., Lyu L. (2019). Chromosome level draft genomes of the fall armyworm, *Spodoptera frugiperda* (Lepidoptera: Noctuidae), an alien invasive pest in China. BioRxiv.

[60] Liu Y., Liu C., Lin K., Wang G. (2013). Functional specificity of sex pheromone receptors in the cotton bollworm *Helicoverpa armigera*. PLoS ONE.

[61] De Fouchier A., Walker W.B., Montagné N., Steiner C., Binyameen M., Schlyter F., Chertemps T., Maria A., François M.-C., Monsempes C. (2017). Functional evolution of Lepidoptera olfactory receptors revealed by deorphanization of a moth repertoire. Nat. Commun..

[62] Bastin-Héline L., De Fouchier A., Cao S., Koutroumpa F., Caballero-Vidal G., Robakiewicz S., Monsempes C., François M.-C., Ribeyre T., Maria A. (2019). A novel lineage of candidate pheromone receptors for sex communication in moths. eLife.

[63] Dong S., Du J. (2002). Chemical identification and field tests of sex pheromone of beet armyworm *Spodoptera exigua*. Acta Phytophyl. Sin..

[64] Wakamura S. (1987). Sex pheromone of the beet armyworm, *Spodoptera exigua* (Huebner) (Lepidoptera: Noctuidae): Field attraction of male moths in Japan to (Z, E)-9, 12-tetradecadienyl acetate and (Z)-9-tetradecen-1-ol. Appl. Entomol. Zool..

[65] Jung C.R., Park Y.J., Boo K.S. (2003). Optimal sex pheromone composition for monitoring *Spodoptera exigua* (Lepidoptera: Noctuidae) in Korea. J. Asia Pac. Entomol..

[66] Tamaki Y., Noguchi H., Yushima T. (1973). Sex pheromone of *Spodoptera litura* (F.) (Lepidoptera: Noctuidae): Isolation, identification, and synthesis. Appl. Entomol. Zool..

[67] Heppner J. (1998). *Spodoptera* armyworms in Florida (Lepidoptera: Noctuidae). Entomol. Circ..

[68] Yan Q., Liu X.-L., Wang Y.-L., Tang X.-Q., Shen Z.-J., Dong S.-L., Deng J.-Y. (2019). Two sympatric *Spodoptera* species could mutually recognize sex pheromone components for behavioral isolation. Front. Physiol..

[69] De Fouchier A., Sun X., Monsempes C., Mirabeau O., Jacquin-Joly E., Montagné N. (2015). Evolution of two receptors detecting the same pheromone compound in crop pest moths of the genus *Spodoptera*. Front. Ecol. Evol..

[70] Malo E.A., Castrejón-Gómez V.R., Cruz-López L., Rojas J.C. (2004). Antennal sensilla and electrophysiological response of male and female *Spodoptera frugiperda* (Lepidoptera: Noctuidae) to conspecific sex pheromone and plant odors. Ann. Entomol. Soc. Am..

[71] Batista-Pereira L.G., Stein K., De Paula A.F., Moreira J.A., Cruz I., Figueiredo M.D.L.C., Perri J., Corrêa A.G. (2006). Isolation, identification, synthesis, and field evaluation of the sex pheromone of the Brazilian population of *Spodoptera frugiperda*. J. Chem. Ecol..

[72] Chang H., Liu Y., Yang T., Pelosi P., Dong S., Wang G. (2015). Pheromone binding proteins enhance the sensitivity of olfactory receptors to sex pheromones in *Chilo suppressalis*. Sci. Rep..

[73] Große-Wilde E., Gohl T., Bouché E., Breer H., Krieger J. (2007). Candidate pheromone receptors provide the basis for the response of distinct antennal neurons to pheromonal compounds. Eur. J. Neurosci..

[74] Pregitzer P., Greschista M., Breer H., Krieger J. (2014). The sensory neurone membrane protein SNMP1 contributes to the sensitivity of a pheromone detection system. Insect Mol. Biol..

[75] Li Z., Ni J.D., Huang J., Montell C. (2014). Requirement for *Drosophila* SNMP1 for rapid activation and termination of pheromone-induced activity. PLoS Genet..

