# Supplementary material for: Functional Characterization of Sex Pheromone Receptors in the Fall Armyworm (Spodoptera frugiperda)
Source: Insects. 2020 Mar 18;11(3):193. doi: 10.3390/insects11030193 (PMC7143582; doi:10.3390/insects11030193)
Supplement: Supplementary file 1 [file insects-11-00193-s001.pdf]

Supplementary Materials

Table 1. Primers used in this study.

Primer name	Sequence (5´- 3´)
Specific primers for cloning	
SfruOR6-F	ATGGGTCTTAAACACTTTCTCTTTGA
SfruOR6-R	TCAAATGCTGCGTAAGAAGGTG
SfruOR11-F	ATGTTTAGCTTGGACACGTATGCG
SfruOR11-R	TTAAAATGTACGTAAGAAAGCGAAATAT
SfruOR13-F	ATGGACGACATAAAATTGTCTACCG
SfruOR13-R	TTATTCTTCCTCGTCGGCGA
SfruOR16-F	ATGAATCTCAAAAAATTCCTTTTCG
SfruOR16-R	TCACATGCTTCTCAAGAAGGTGA
SfruOR56-F	ATGGGTTTAAGAAATTTTCTTTTTGG
SfruOR56-R	TCAGATGCTACGTAAAAAGGTGAAA
SfruOR62-F	ATGGGTCTCAAAAAATTCCTATTCG
SfruOR62-R	TCAGATGCTGCGTAAAAACGC
SfruORco-F	ATGATGACCAAAGTGAAAGCCC
SfruORco-R	TTACTTGAGCTGTACTAACACCATGAA
Primers for Real-time PCR	
SfruOR6-qF	TGCTGACCCATACCTTGCAC
SfruOR6-qR	CAGTAACAGCAAACAGCCGC
SfruOR11-qF	ACGCTGTCGTAGGCATCACC
SfruOR11-qR	TTCTCGCCTAGCGCCTTTCC
SfruOR13-qF	TACAGCGTGCCCTGGGAGTA
SfruOR13-qR	TGGTCTGGACACCGACAGTGA
SfruOR16-qF	TGGGAATGCATGGATGCGAACA
SfruOR16-qR	TCGCGGCCATAGAAGTGACG
SfruOR56-qF	GCTCCAGCCCTTATGCGTTA
SfruOR56-qR	GACGCCGACATTGGCAATAC
SfruOR62-qF	GAAGTGGGGGCACACAAAAC
SfruOR62-qR	GCAGAACCAAGTAGCGCAGA
SfruRpL32-qF	GACCCGTCACATGCTACCCA
SfruRpL32-qR	GCGCGCTCTACGATGGTCTT
Sfru EF1α-qF	ACGCTCCCGGACACAGAGAT
Sfru EF1α-qR	AGCGGCGACAATGAGTACGG
Primers for cRNA synthesis	
SfruOR6-EcoRI-F	ATTCCCCGGGGATCC***GAATTC***ATGGGTCTTAAACACTTTCTCTTTGA
SfruOR6-XbaI-R	TCGGCGATCGGGCCC***TCTAGA***TCAAATGCTGCGTAAGAAGGTG
SfruOR11-EcoRI-F	ATTCCCCGGGGATCC***GAATTC***ATGTTTAGCTTGGACACGTATGCG
SfruOR11-XbaI-R	TCGGCGATCGGGCCC***TCTAGA***TTAAAATGTACGTAAGAAAGCGAAATA<br>T
SfruOR13-EcoRI-F	ATTCCCCGGGGATCC***GAATTC***ATGGACGACATAAAATTGTCTACCG
SfruOR13-XbaI-R	TCGGCGATCGGGCCC***TCTAGA***TTATTCTTCCTCGTCGGCGA
SfruOR16-EcoRI-F	ATTCCCCGGGGATCC***GAATTC***ATGAATCTCAAAAAATTCCTTTTCG
SfruOR16-XbaI-R	TCGGCGATCGGGCCC***TCTAGA***TCACATGCTTCTCAAGAAGGTGA
SfruOR56-EcoRI-F	ATTCCCCGGGGATCC***GAATTC***ATGGGTTTAAGAAATTTTCTTTTTGG
SfruOR56-XbaI-R	TCGGCGATCGGGCCC***TCTAGA***TCAGATGCTACGTAAAAAGGTGAAA
SfruOR62-EcoRI-F	ATTCCCCGGGGATCC***GAATTC***ATGGGTCTCAAAAAATTCCTATTCG
SfruOR62-XbaI-R	TCGGCGATCGGGCCC***TCTAGA***TCAGATGCTGCGTAAAAACGC

SfruORco-EcoRI-F	ATTCCCCGGGGATCC***GAATTC***ATGATGACCAAAGTGAAAGCCC
SfruORco-XbaI-R	TCGGCGATCGGGCCC***TCTAGA***TTACTTGAGCTGTACTAACACCATGAA

Italic sequences (*GAATTC* and *TCTAGA*) are the restriction sites of *EcoRI* and *XbaI*, respectively; and the underlined sequences are homologs to the sequences of pGH19.

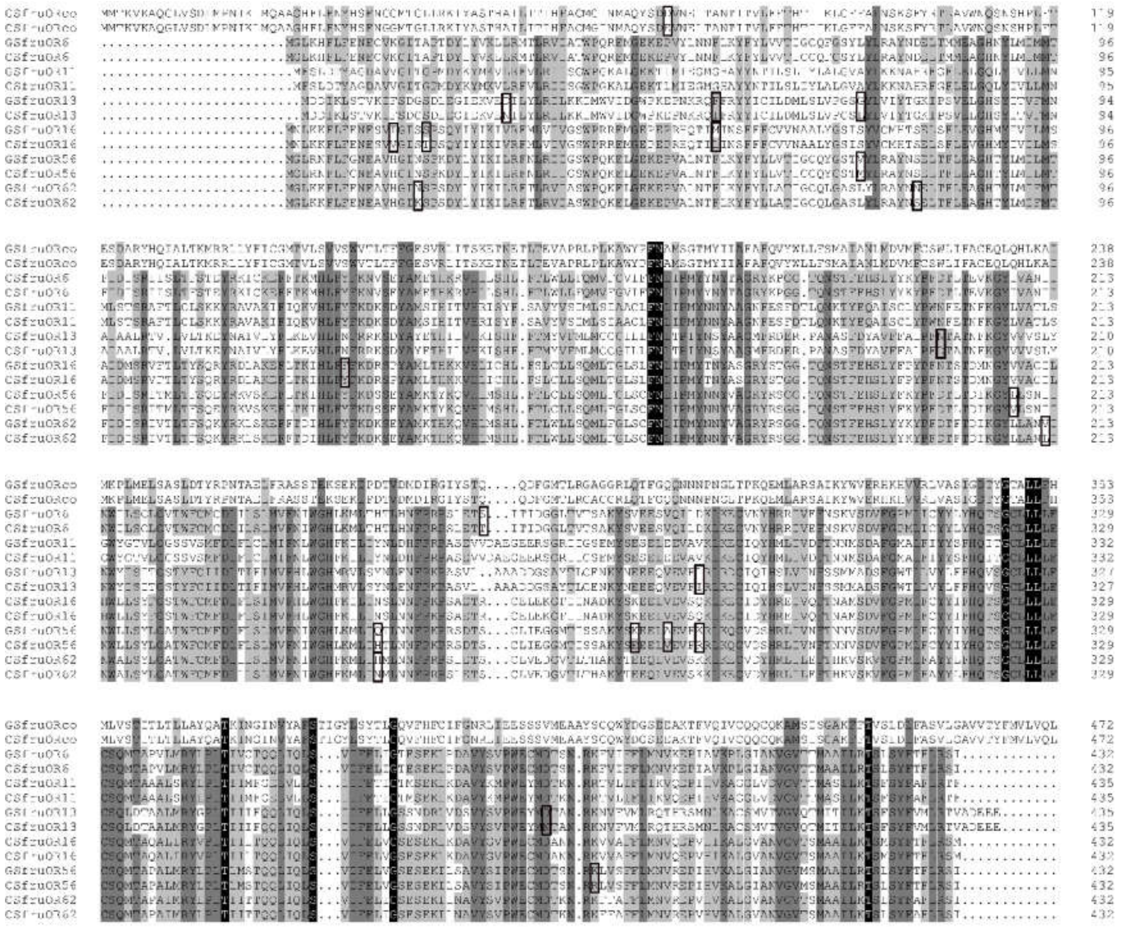

Figure S1. Alignment of the reported PR sequences obtained by genome sequence analysis (GSfruORs) with the sequences cloned in the present study (CSfruORs). Three to five positive clones of each gene were selected and sequenced, and the clones of each gene were identical. The amino acids differences of the proteins between GSfruORs and CSfruORs are marked with a black frame.

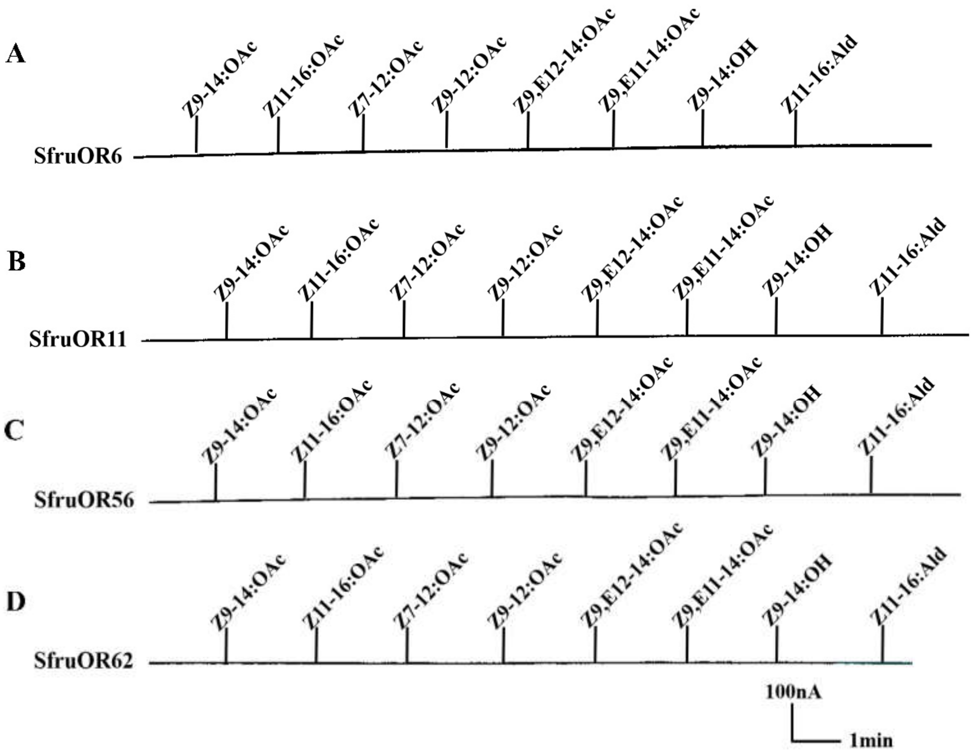

Figure S2. Responses of *Xenopus* oocytes co-expressed with SfruOR6/ORco **(A)**, SfruOR11/ORco **(B)**, SfruOR56/ORco **(C)** and SfruOR62/ORco **(D)** to stimulation with pheromone components and analogs (10^{-4} M).
